# Balint Group Sessions for Medical Students, a Pilot Study

**DOI:** 10.1192/bjo.2022.147

**Published:** 2022-06-20

**Authors:** Annette Ros, Abby Older, Eleanor Gaynor, Bayode Shittu, Christopher Bu, Kathia Sullivan

**Affiliations:** Cheshire and Wirral Partnership NHS Foundation Trust, Merseyside, United Kingdom

## Abstract

**Aims:**

Core trainees in psychiatry all complete a year of Balint group sessions. These sessions are invaluable, as improved awareness of our own thoughts and feelings is a crucial part of our development as clinicians. We considered that it may have been helpful to have started these sessions at an earlier stage of medical training, for example, in medical school.

**Methods:**

We approached the University of Liverpool School of Medicine and proposed a pilot Balint programme with 4th year medical students rotating through psychiatry in Cheshire Wirral Partnership Trust.

Sessions were conducted in 4-week blocks, corresponding with the students’ psychiatry rotations. To allow sufficient time for all students to contribute in each hour-long session, groups were limited to a maximum of 7 students. Each group was allocated 2 facilitators and was conducted on Microsoft Teams because of COVID-19-related restrictions. Facilitators had fortnightly supervision with a consultant psychotherapist.

At the end of each 4-week block, anonymous feedback was collected. Small alterations were made to the programme during the course of the pilot in response to attendance rates, punctuality and feedback.

**Results:**

18 (approximately 50%) of the students from the first 3 cohorts submitted feedback:

- All said the experience helped them reflect more on their interactions with patients and colleagues and improved their insight into how others think/feel in caring for patients.
- 94% said they enjoyed it; they thought they would use the skills they had developed; and they would participate again in future if given the option.
- 83% said 4 sessions was ‘just right’, 11% said ‘not enough’ and 6% said ‘maybe too much’;
- 72% rated their overall experience of the programme as ‘excellent,’ 17% as ‘good,’ 11% as ‘fair’.

Free-text feedback was positive. Students valued the opportunity to reflect on the emotions and interpersonal dynamics experienced in clinical scenarios. Critical feedback was mostly around a preference to have sessions face-to-face and a desire to have more sessions.

As facilitators, the experience has helped us increase our reflective capacity and gain confidence in leading, managing group dynamics and setting boundaries.

**Conclusion:**

Student experience of the Balint programme was positive for the majority. From a facilitator perspective, we found the experience rewarding and beneficial for professional development. Currently only approximately 1/3 students rotate through this trust and can therefore benefit from the sessions. This pilot study provides supporting evidence for extending the scheme to all 4th year Liverpool University medical students.

